# Supplementary material for: Effect of a Cross-Training and Resistance Exercise Routine on IL-15 in Adults with Type B Acute Lymphoblastic Leukemia during the Induction Phase: Randomized Pilot Study
Source: J Funct Morphol Kinesiol. 2023 Dec 21;9(1):4. doi: 10.3390/jfmk9010004 (PMC10970970; doi:10.3390/jfmk9010004)
Supplement: Supplementary file 1 [file jfmk-09-00004-s001.zip › jfmk-2727894-supplementary.pdf]

Journal of
Functional Morphology
and Kinesiology

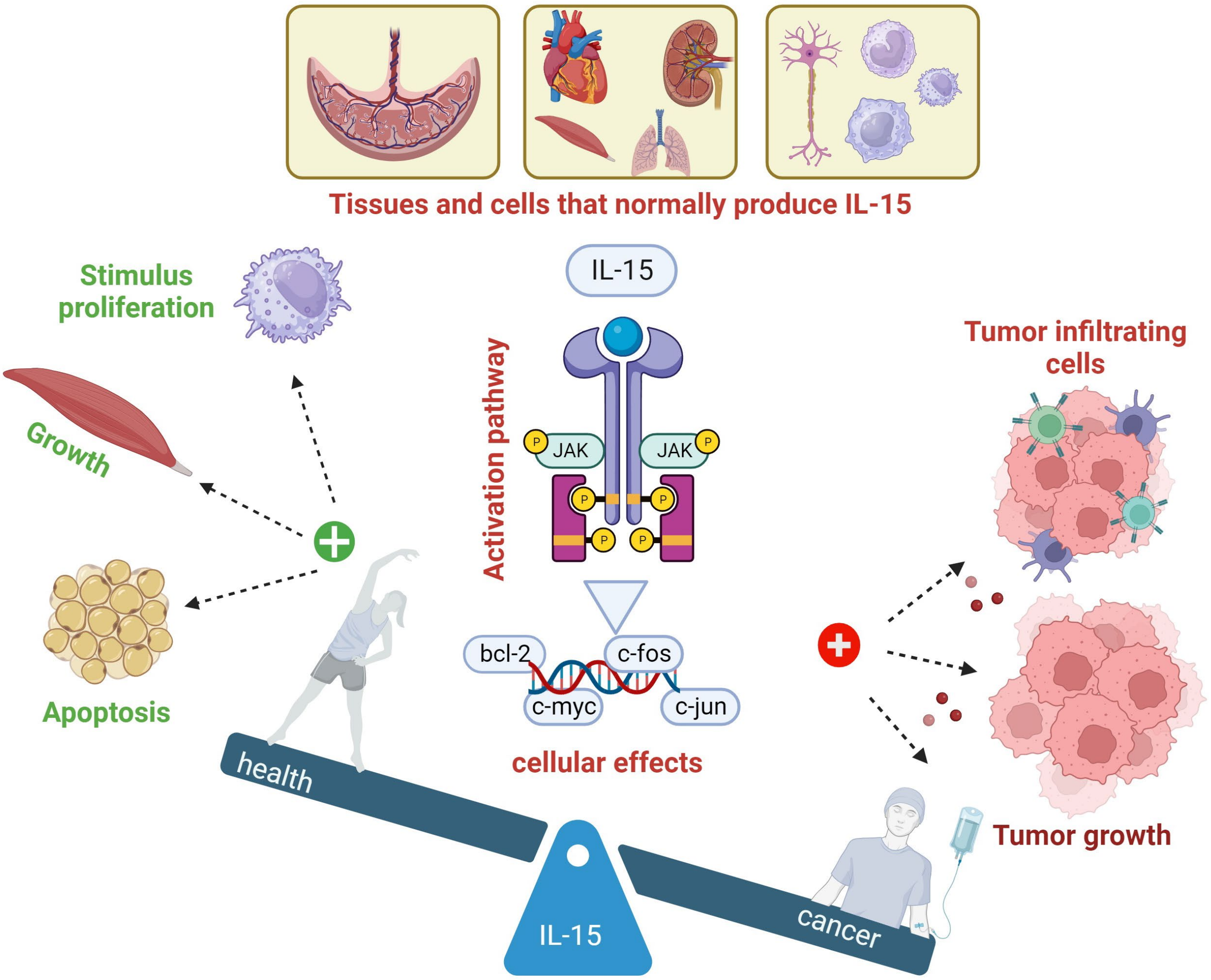

Figure S1. The role of IL-15 in energy metabolism, muscle growth, and its relationship with cancer and cellular components.
